# A Tet-Inducible CRISPR Platform for High-Fidelity Editing of Human Pluripotent Stem Cells

**DOI:** 10.3390/genes13122363

**Published:** 2022-12-14

**Authors:** Shawna L. Jurlina, Melissa K. Jones, Devansh Agarwal, Diana V. De La Toba, Netra Kambli, Fei Su, Heather M. Martin, Ryan Anderson, Ryan M. Wong, Justin Seid, Saisantosh V. Attaluri, Melissa Chow, Karl J. Wahlin

**Affiliations:** 1 Viterbi Family Department of Ophthalmology, Shiley Eye Institute, University of California San Diego, La Jolla, CA 92093, USA; 2 Department of Bioengineering, University of California San Diego, La Jolla, CA 92093, USA; 3 Department of Biotechnology, California State University Channel Islands, Camarillo, CA 93012, USA; 4 Department of Biology, California State University San Marcos, San Marcos, CA 92096, USA

**Keywords:** CRISPR, Cas9, HDR, homology-directed repair, gene-editing, DTS, transfection, stem cell, iCas9

## Abstract

Pluripotent stem cells (PSCs) offer an exciting resource for probing human biology; however, gene-editing efficiency remains relatively low in many cell types, including stem cells. Gene-editing using the CRISPR-Cas9 system offers an attractive solution that improves upon previous gene-editing approaches; however, like other technologies, off-target mutagenesis remains a concern. High-fidelity Cas9 variants greatly reduce off-target mutagenesis and offer a solution to this problem. To evaluate their utility as part of a cell-based gene-editing platform, human PSC lines were generated with a high-fidelity (HF) tetracycline-inducible engineered *Streptococcus pyogenes* SpCas9 (HF-iCas9) integrated into the AAVS1 safe harbor locus. By engineering cells with controllable expression of Cas9, we eliminated the need to include a large Cas9-expressing plasmid during cell transfection. Delivery of genetic cargo was further optimized by packaging DNA targeting guide RNAs (gRNAs) and donor fragments into a single plasmid backbone. The potential of homology-directed repair (HDR) based gene knock-in at the *CLYBL* safe harbor site and endogenous *SOX2* and *SIX6* genes were demonstrated. Moreover, we used non-homologous end-joining (NHEJ) for gene knockout of disease-relevant alleles. These high-fidelity CRISPR tools and the resulting HF-iCas9 cell lines will facilitate the production of cell-type reporters and mutants across different genetic backgrounds.

## 1. Introduction

Originally identified in bacteria, the Clustered Regularly Interspaced Palindromic Repeat (CRISPR) Cas9 gene editing system has since been repurposed for the precision editing of DNA from a wide array of eukaryotic cells, including human stem cell lines [1,2,3,4]. An obstacle to efficient gene editing has been variable targeting efficiency and potential off-target effects that can introduce unwanted mutations. Although efforts to improve the efficiency of homology-directed repair (HDR) for precision gene knock-in and non-homologous end joining (NHEJ) for mutagenesis have been partially successful using small molecule chemical inhibitors, the success of this approach varies and can be difficult to reproduce. Optimization of plasmid size, sequence composition and targeting may also lead to improved gene-editing efficiency. In this regard, the development of enhanced specificity *Streptococcus pyogenes* Cas9 (eSpCas9) via structure-guided mutagenesis has greatly reduced the number of off-target effects while maintaining robust on-target cleavage activity, thereby proving its utility in gene editing applications requiring increased levels of specificity [5].

Previous reports suggested that plasmid DNA uptake into the nucleus can be facilitated by a smaller plasmid size, such as with mini-circle technology, or through the use of small DNA-targeting sequences (DTS), which may increase plasmid nuclear entry by co-transport with endogenous transcription factors [6,7]. For example, hypoxia-inducible factor (HIF) is reported to orchestrate the expression of more than 50 genes related to hypoxia [8,9,10] and the introduction of a hypoxia-responsive element (HRE) or DTS sequence increased the rate of plasmid nuclear transport under low oxygen [11]. Under hypoxia, a stabilized HIF-1 α protein dimerizes with its β subunits and activates genes with hypoxia-responsive promoter elements (HRE). In theory, plasmids with HRE elements could piggyback onto transcription factors as they move into the nucleus. While previous studies have demonstrated that DTS components can influence transfection efficiency, this concept has not been broadly applied to plasmid design, particularly for gene editing in human PSCs.

The reproducibility and robustness of stem cell models to study human development and disease are limited by the labor required to generate reporter and mutant cell lines. A cell-based gene-editing pipeline could address this by facilitating a more efficient generation of cell-based reporters and disease-relevant mutations, respectively. Towards this goal, tetracycline (Tet) inducible high-fidelity eSpCas9 (HF-iCas9) and constitutive rtTA cassettes were integrated into different alleles at the AAVS1 safe harbor site in PSCs from different genetic backgrounds. These work in tandem with a simplified hybrid donor that expresses a U6-driven DNA targeting guide RNA (gRNA) and a DNA donor template for HDR [5]. We utilized eSpCas9 bearing the K848A, K1003A and R1060A mutations since this variant has undetectable off-target indel formation and high on-target activity. This hybrid vector also incorporates minicircle compatible attP and attB øC31-integrase sites for recombination and I-SceI homing-endonuclease sites for degradation of unwanted bacterial parental plasmid sequences [12]. Cumulatively, this system offers several advantages, it: (1) utilizes a Tet-inducible engineered HF-iCas9 protein to minimize unwanted off-target mutagenesis, (2) uses a single hybrid vector for delivery of both the gRNA and DNA donor, (3) has the potential to generate a smaller footprint with less bacterial plasmid DNA, and (4) utilizes a more effective gRNA scaffold that is optimized for genome-editing by HF-iCas9. To demonstrate the broad applicability of these tools, we carried out gene-targeting at the *CLYBL* safe harbor site [13,14], the endogenous genes *SOX2*, which is active in stem cells, and *SIX6*, which is active during eye-field, pituitary and hypothalamic tissue fate specification. *SIX6*-GFP recombinant lines were generated in three different genetic backgrounds and were demonstrated to retain the capability to develop into optic vesicles. Lastly, we used these lines to create loss-of-function retinal disease model stem cells through NHEJ insertion/deletion (indel) formation. Overall, this platform will accelerate the creation of fluorescent reporters for studies of neural development and mutant cells for disease modeling.

## 2. Materials and Methods

### 2.1. Pluripotent Stem Cells

IMR90.4 iPSCs and WA09 ESCs (both from WiCell, Madison, WI, USA) and EP1.1 iPSCs (Johns Hopkins University, Baltimore, MD, USA) were used for the following study [15,16,17]. Cells were routinely tested for mycoplasma by PCR [18]. PSCs were used with approval from the UC San Diego Institutional Review Board (IRB).

### 2.2. Single-Cell Passage and Maintenance of PSCs

Stem cells were maintained antibiotic-free on 1% (*vol/vol*) Matrigel (MG)-GFR™ (#354230; Corning, New York, NY, USA) extracellular matrix (ECM) coated dishes at 37 °C under hypoxic conditions (10% CO_2_/5%O_2_) in mTeSR1 (Stem Cell Technologies, Vancouver, Canada) as previously described [19,20,21,22]. Cells were passaged every 4–6 days, with Accutase (#A6964; Sigma-Aldrich, St. Louis, MO, USA) for 8–10 min, dissociated into single cells, quenched with mTeSR1 plus 5 μM blebbistatin (B; #B0560; Sigma-Aldrich, St. Louis, MO, USA), pelleted at 80× *g* for 5 min, resuspended in mTeSR1+B and plated at 2000 cells per single well of a 12-well plate [23]. After 48 h, cells were fed with mTeSR1 alone. To ensure cell quality, chromosomal integrity was evaluated by copy number variation (CNV) analysis using an Infinium HumanCore-24 v1.1 BeadChip (Illumina, San Diego, CA, USA).

### 2.3. Cloning

See Supplementary Tables S1 and S4 for a complete list of oligos.

### 2.4. Constructs for Generating Tet-Inducible HF-iCas9 Cells

To create HF-ieCas9 cells we targeted the AAVS1 safe harbor site inserting a reverse tetracycline-controlled trans-activator (rtTA) expression cassette at one allele and a 3G-Tet-regulated eSpCas9 (HF-iCas9) [5] at the other. When bound to doxycycline (dox), constitutively expressed rtTA binds to the Tet operator and ieCas9 expression is induced. The rtTA coding sequence was provided by the AAVS1-Neo-M2-rtTA plasmid (Addgene #60843) [24] whereas the HF-iCas9 coding sequence was amplified from eSpCas9(1.1)(Addgene #71814) [5] and cloned into the AAVS1-puro-Cas9 donor (Addgene #58409) [25]. Briefly, the AAVS1 donor shell was amplified with Phusion Polymerase (#F548L; Thermo Fisher Scientific, Waltham, MA, USA) using the oligos Cas9backbone_F and Rev and the HF-iCas9 insert was amplified using the oligos Cas9insert_for and Cas9insert_rev (Table S1). PCR products were purified using DNA Clean and Concentrator-5 columns (#D4014; Zymo Research, Irvine, CA, USA) and donor and insert PCR products were fused together using Gibson assembly (#E2611S; NEB, Ipswich, MA, USA), followed by overnight digestion with Dpn1 enzyme (#R0176L; NEB, Ipswich, MA, USA) to remove unwanted parental template DNA. The Gibson product was transformed into chemically competent Stable *E. coli* cells (#C3040H, NEB, Ipswich, MA, USA) and colonies were miniprepped and verified by Sanger sequencing. For AAVS1 targeting, a phosphorylated oligo duplex of the AAVS1_esp_T1 forward and reverse oligos were ligated into BbsI cut pSpCas9(BB)-2A-puro (PX459) V2.0 backbone (Addgene #62988) [4] using Quick ligase (#M2200L; NEB, Ipswich, MA, USA) according to the manufacturer’s specifications).

### 2.5. Donor Plasmids for Gene-Targeting

Geneious Prime software (Biomatters, Auckland, New Zealand) was used to identify high off-target score CRISPR targeting sites near the *SOX2* and *SIX6* stop sites as well as the *CLYBL* safe harbor site (Table S2). PCR amplifications were carried out with Phusion Flash Polymerase (#F548L; Thermo Fisher Scientific, Waltham, MA, USA) and appropriate oligos (Tables S1 and S4). PCR products were column purified using DNA Clean and Concentrator-5 columns (#D4014; Zymo Research, Irvine, CA, USA). DNA fragments were stitched together using HiFi DNA Assembly Mastermix (#E2621S; NEB, Ipswich, MA, USA), and parental plasmids were removed with the methylation-sensitive Dpn1 enzyme (#R0176L; NEB, Ipswich, MA, USA). For all work except for minicircle production, plasmids were transformed into NEB Stable Competent *E. coli* (#C3040I; NEB, Ipswich, MA, USA) cells.

#### 2.5.1. Hybrid Donor-Guide RNA Plasmid (Hybrid Donor)

A custom mini-circle compatible hybrid donor plasmid (MC-donor) was constructed by overlap extension PCR and Gibson HiFi assembly (#E2621L; NEB, Ipswich, MA, USA) by incorporating a pUC origin of replication, an ampicillin resistance gene, a docking site for the DNA donor fragment and a human U6-gRNA-scaffold cassette. AttB and attP integrase recombination sites were introduced to facilitate minicircle plasmid production and 2× SceI endonuclease sites were introduced for degradation of parental plasmid after mini-circle induction. Briefly, a cassette containing the hU6 promoter, gRNA target insertion site, and gRNA scaffold was PCR amplified from eSpCas9(v1.1)(Addgene #71814) [5] (Tables S1 and S4). An SV40 polyA DTS site was designed to flank the U6 scaffold.

#### 2.5.2. SOX2-GFP Construct

A 20 bp *SOX2* targeting sequence (GGCCCTCACATGTGTGAGAG) was inserted into the U6 scaffold by site-directed mutagenesis using Gibson assembly followed by digestion with the methylation-sensitive Dpn1 enzyme. A *SOX2* donor fragment (663 bp upstream/710 bp downstream homology arms) was then PCR amplified from EP1.1 iPSC genomic DNA and cloned into the plasmid backbone, also by Gibson assembly. Immediately before the stop codon, a porcine teschovirus 2A (p2A) polycistronic sequence was stitched in-frame with enhanced GFP (GFP) fused to an SV40 nuclear localization signal (nls). For experiments requiring different DTS sequences, site-directed mutagenesis was used to swap out SV40 for HRE DTS sequences.

#### 2.5.3. CLYBL-Cbh-mRuby3 Construct

For constitutive expression of mRuby3 at the *CLYBL* safe harbor site (SHS) we first swapped in a 20 bp *CLYBL* targeting sequence (GACCATACTATCTAGAAATA) between the human U6 promoter and the guide RNA scaffold. Next, we sequentially amplified and inserted a fragment of human genomic DNA flanking the *CLYBL* target site (690 bp left/590 right homology arms) and a Cbh-mRuby3 expression cassette which was inserted between the left and right homology arms.

#### 2.5.4. SIX6-GFP Construct

For the *SIX6*-GFP plasmid, we swapped in a 20 bp SIX6 targeting sequence (GACATCTGAGTTGCCCATCC) after the U6 promoter by site-directed mutagenesis, then amplified a 1311 base pair donor arm (657 left arm and 654 base right arm) from human genomic DNA and inserted a p2A-h2b-eGFP cassette in-frame just before the stop codon. For gRNA construction, targeting was directed at the underlined stop codon of *SIX6*.

### 2.6. Constructs for Genetic Perturbation

For gene knockout, *AIPL1*, *CRB1* and *RPGRIP1* guides were designed as described above. Guide RNA sequences were inserted by site-directed mutagenesis into the hybrid donor with no homology arms.

### 2.7. Minicircle Production

ZYCY10P3S2T minicircle producer strain *E. coli* cells (#MN900A-1; System Biosciences, Palo Alto, CA, USA) were transformed with parental plasmids (PP) and grown on kanamycin plates. Colonies were picked and grown in 6 mL of lysogeny broth (LB) with kanamycin for 6 h at 37 °C, followed by growth in terrific broth (TB) for 12 h. For øC31 integrase induction, 100 mL of LB broth together with 100 µL of 20% arabinose induction solution (System Biosciences, Palo Alto, CA, USA) and 4 mL of 1 N NaOH was added to the culture and grown for 5.5 h at 30 °C. Endotoxin-free plasmids were prepared using the PureLink plasmid Midiprep kit (#MN900A-1; Thermo Fisher Scientific, Waltham, MA, USA) to purify PP and MC. PP contamination was removed using the Plasmid Safe ATP-dependent DNase kit (#E3110K; Lucigen, Middleton, WI, USA), and the products were purified using the DNA Clean & Concentrator-25 kit (#D4033, Zymo Research, Irvine, CA, USA). Verification of plasmid cutting was carried out using Kpn1 or Ase1 restriction enzyme in CutSmart buffer at 37 °C for 5 h followed by heat inactivation for 20 min at 65 °C.

### 2.8. Plasmid Preparation for Transfection

For routine growth of plasmids, we used chemically competent Stable *E. coli* cells (#C3040I; NEB, Ipswich, MA, USA). DNA for transfection was prepared using the endotoxin-free grade Plasmid Plus Maxi Kit (#12963; Qiagen, Hilden, Germany) or ZymoPURE Plasmid Midiprep Kits (#11-550; Zymo Research, Irvine, CA, USA). The purified plasmid was resolved by agarose gel electrophoresis to rule out RNA or genomic DNA contamination. Plasmids used for quantitative studies of HDR efficiency were never thawed more than 3 times to prevent unwanted degradation of plasmids.

### 2.9. Generation of Tet-Inducible Cell Lines

To generate tetracycline-inducible lines, PSCs were enzymatically treated with Accutase for 12 min at 37 °C, quenched in mTeSR1 with blebbistatin (B), then 200,000 iPSCs were centrifuged at 80× *g* for 5 min and media was then removed. Cells were resuspended in 10 µL’s of R-buffer containing 1 µg of AAVS1-ieCas9 donor, 1 µg AAVS1-Neo-M2-rtTA donor, 1 µg of eSpCas9 (v1.1) expression vector and 0.5 µg AAVS1gT1 gRNA vector, and immediately electroporated with a Neon transfection system (#MPK5000; Thermo Fisher Scientific, Waltham, MA, USA) with the following settings: (1300 V, 20 ms, 1 pulse), plated into mTeSR1+B on Matrigel-coated plates and transferred to a 37 °C hypoxia (5%O_2_/10%CO_2_) incubator. Cells were treated with 500 ng/mL puromycin (puro) three days after transfection for an additional 3 days, passaged, and then treated with puro for an additional three days. 50 µg/mL of G418 was then used for secondary selection and single-cell derived clones were expanded and verified for dox inducible HF-iCas9 expression by immunohistochemistry (see below).

### 2.10. Confirmation of HF-iCas9 Inducible Clones

To verify the functionality of puro/G418 selected cells containing both HF-iCas9 and 3G-rtTA cassettes, we passaged cells to single cells and transferred the resulting colonies into Matrigel-coated 48-well TC treated plates. Briefly, a cell scraper (#89260, VWR, Radnor, PA, USA) was used to lift colonies, and an inverted microscope (Leica DM1) at 4× magnification was used to assist transfer with a P20 pipet. Colonies were expanded and replica plated with one of the plates receiving 1 μg/mL dox to drive HF-iCas9 expression. For immunocytochemistry (ICC), cells were fixed for 5 min in 4% PFA solution in phosphate-buffered saline (PBS) containing 5% sucrose and rinsed 3 times with PBS for 5 min each. A mouse Cas9-specific monoclonal antibody (clone 7A9-3A3 #14697; Cell Signaling Technology, Danvers, MN, USA) was diluted at 1:800 in PBS containing 2% horse serum and 0.1% Triton X-100 and incubated overnight at 4 °C. Cells were rinsed thrice with PBS and ICC signals were detected after incubation in a 1:1000 dilution of donkey anti-mouse Alexa Fluor-488 antibody (#A21202; Thermo Fisher Scientific, Waltham, MA, USA) followed by 3 washes in PBS. Cells were counterstained with 10 μg/mL 4’, 6-diamidino-2-phenylindole (DAPI; #10236276001; Roche, Basel, Switzerland) to identify cell nuclei and imaged using a Zeiss Axio Observer Z1. For expansion, we chose only replica-plated clones with a homogenous AlexaFluor-488 signal indicating functional expression of both rtTA and Cas9.

cDNA was prepared from 0 to 3-day dox treated HF-iCas9 PSC RNA using the Bio-Rad iScript Reverse Transcription Super Mix for qRT-PCR (#1709941; Bio-Rad, Hercules, CA, USA). qRT-PCR was performed on a Bio-Rad CFX Connect Real-Time PCR Detection System (Bio-Rad, Hercules, CA, USA) using Bio-Rad SYBR Green Supermix (Bio-Rad, Hercules, CA, USA). Biological replicates (*n* = 3) were analyzed in technical duplicates and normalized to the expression of the reference gene hRPL27, and relative expression was determined by the ∆Ct method. Briefly, the ∆Ct value was determined by subtracting the Ct of the reference gene from the Ct of the gene of interest (hCas9) at various time points. The relative fold change gene expression was calculated as per the Equation 2^-(∆Ct). Independently, we also evaluated the absolute expression of hCas9 using a standard curve method. A plot was generated between Cq and known starting quantities (in pg) of hCas9 spanning a 128-fold serial dilution. A linear fit was applied to the graph and verified to have R^2 > 0.98. The equation from the fit was then used to calculate the starting quantities of hCas9 in doxycycline-treated HF-iCas9 PSCs across days 0 to 3. The following primers were used: Cas9_137R: GGTGCTGGTGTACCTCTTCC, Cas9_137F: ACAACAAGCACCGGGATAAG, RPL27_123R: TCTGAAGACATCCTTATTGACG, RPL27_123F: ATCGCCAAGAGATCAAAGATAA, RPL30_158_R: AAAGGAAAATTTTGCAGGTT, RPL30_158_F: ACAGCATGCGGAAAATACTAC.

### 2.11. Generation of Reporter Lines

Tet-inducible HF-iCas9 PSCs were treated overnight with 1 μg/mL dox and transfected with 1–4 µg of reporter donor plasmids (SOX2-GFP, SIX6-GFP, CLYBL-Cbh-mRuby3 constructs). SOX2-GFP and CLYBL-mRuby3 modified colonies were selected one-week post-transfection since by then only stably integrated cells will retain fluorescence. As above, clones were gently scraped and manually transferred to Matrigel-coated 48-well plates and stably integrated cells were identified by fluorescence one day later. *SIX6*- GFP cells, which are not fluorescent until after differentiation, were selected by a similar approach and genotyped by PCR (see Genotyping below). Since most harvested colonies were initially heterogeneous, each positive colony was selected a second time and re-genotyped to ensure homogeneity.

### 2.12. Microscopy and Image Analysis

SOX2-GFP+ cell counts were measured as a function of total cell nuclei labeled with DRAQ5 (#62251; Thermo Fisher Scientific, Waltham, MA, USA) or Hoechst 33342 (#H1399; Thermo Fisher Scientific, Waltham, MA, USA) nuclear counterstains at concentrations of 10 µM and 10 μg/mL, respectively, for 20 min at 37 °C. DRAQ5 stained samples were washed 6 times with pre-warmed 1 × PBS (#14040; Thermo Fisher Scientific, Waltham, MA, USA) followed by FluoroBrite DMEM (#A1896702; Thermo Fisher Scientific, Waltham, MA, USA) which increased the signal-to-noise ratio of fluorescent signals. Hoechst-stained samples were rinsed with fresh media. To reduce background fluorescence, we visualized fluorescent cells in a reduced volume of cell culture medium. For quantification, images were acquired with an ImageXpress Micro Confocal High-Content microscope using custom modules in the MetaXpress software package (Molecular Devices, San Jose, CA, USA). The percentage of GFP+ was determined by defining Hoechst+ objects as the total cell numbers and dual GFP/Hoechst dual + as % converted. A small percentage of dead or dying cells with green autofluorescence was detected by automated cell counting, however, this contribution was minimal. Custom module settings used for cell counts were as follows: ‘count GFP nuclei objects’: min. object width 7 μm, max. object width 11 μm, intensity above threshold 89; ‘count Hoechst nuclei objects’: min. object width 8 μm, max. object width 12 μm, intensity above threshold 126. Measurement inputs were set at a standard area value of 10 μm.

### 2.13. Statistical Analysis

Results were quantified using Prism (v9, GraphPad Software, San Diego, CA, USA). One-way ANOVA was used to test statistical significance and for multiple comparison tests, a Dunnett’s multiple comparison was used with an α cutoff of 0.05. For comparisons with less than three groups, an unpaired student’s *t*-test was used. Unless otherwise stated in figure legends, data are presented as mean ± SEM and the following symbols are used to represent *p* values, * *p* < 0.05, ** *p* < 0.01, *** *p* < 0.001, and **** *p* < 0.0001. N represents the number of independent experiments.

### 2.14. NHEJ Based Mutagenesis

To induce in/del formation by NHEJ, we used the same plasmid backbone, including the U6-gRNA-scaffold cassette, that was used for HDR except that these did not contain a donor fragment. Each plasmid included a U6 promoter, a 20 base-pair target sequence and the V1 scaffold lacking the flip in the fifth nucleotide or 5 base pair extensions to enhance the GAAA loop structure [4,26]. To swap in different gRNAs between the U6 promoter and scaffold sequences, we used site-directed mutagenesis using PCR to amplify the shell with gRNA overhangs, followed by Gibson assembly to chew back and ligate overhang sequences. Overnight Dpn1 digestion was carried out to remove the original parental plasmid. Plasmids were confirmed by Sanger sequencing and aligned with the expected sequence in Geneious Prime (Biomatters, Auckland, New Zealand).

### 2.15. Genotyping

Single-cell clones were expanded and genotyped after cell lysis in Quick Extract (QE) Buffer (#QE09050; Lucigen, Middleton, WI, USA). Lysed genomic DNA underwent first-pass PCR to identify inserts using one oligo flanking the left homology arm and one nested within the H2B fragment (hSIX6GenoT_FlankF and hSIX6GenoT_int_R; Table S1). PCR products were sequence verified using oligos pointing inward towards the fluorescent insert (Table S3). A second pass PCR reaction using oligos flanking the insert (hSIX6_GenoT_2763_F and hSIX6_GenoT_2763_R; Table S1) was used to determine hetero- versus homozygosity. We also evaluated modified cell lines for potential off-target mutagenesis by PCR amplification of potential off-target sites (Table S2) identified using the Geneious Prime software that incorporates whole-genome off-target analysis algorithms from the Zhang lab [27].

### 2.16. 3D Directed Differentiation

Optic vesicles were generated as previously described [28] with minor modifications. The cell culture medium used for differentiation was as follows: BE6.2-NIM (B27 + E6 at 2× concentration) (neural induction medium) consists of DMEM (#11965; Thermo Fisher Scientific, Waltham, MA, USA) supplemented with 1% B27 vitamin A (−) (#12587010; Thermo Fisher Scientific, Waltham, MA, USA) and 2× E6 supplement (38.8 mg/L insulin (#11376497001; Roche), +128 mg/L L-ascorbic acid (#A8960; Sigma-Aldrich, St. Louis, MO, USA), 28 μg/L sodium selenite (#S5261; Sigma-Aldrich, St. Louis, MO, USA), 21.4 mg/L transferrin (#T0665; Sigma-Aldrich, St. Louis, MO, USA) and 38.8 mg/L NaHCO_3_). LTR (Long-Term Retina) medium was a 3:1 mix of DMEM:F12 (#11965, #11765; Thermo Fisher Scientific, Waltham, MA, USA) supplemented with 1% B27 (#17504044; Thermo Fisher Scientific, Waltham, MA, USA), 10% heat-inactivated qualified-grade FBS (#16140071; Thermo Fisher Scientific, Waltham, MA, USA), 1 mM sodium pyruvate (#11360; Thermo Fisher Scientific, Waltham, MA, USA), 1× NEAA (#11140; Thermo Fisher Scientific, Waltham, MA, USA), 1× GlutaMAX (#35050061; Thermo Fisher Scientific, Waltham, MA, USA) and 1 mM taurine (#T8691; Sigma-Aldrich, St. Louis, MO, USA). For optic vesicle induction, PSCs maintained in mTeSR1 were used to initiate serum-free embryoid body forced-aggregates (SFEBs). Stem cells were passaged with a longer Accutase incubation for 12 min and 1000 cells in 50 μL of mTeSR1+B were seeded per well into polystyrene 96-well U-bottom plates (#650180; Greiner, Frickenhausen, Germany). Aggregates were transitioned to BE6.2 medium by adding 50 μL + 2% MG on day 1 and 1% MG each day thereafter. On days 4–8, a 50% medium exchange (100 μL) was performed daily and every other day thereafter. The medium contained 1% (*v*/*v*) MG and 3 μM of IWR-1e (a WNT inhibitor and AXIN2 stabilizer; #681669; EMD Millipore, Burlington, VT, USA) from days 1–6. For long-term maintenance, vesicles were transferred at day 10 to 15 mL conical tubes, rinsed 3 times in HBSS, and resuspended in BE6.2 + 300 nM Smoothened agonist (SAG; #566660; EMD Millipore, Burlington, VT, USA) from days 10–12 to enhance retinal induction then LTR + SAG from days 12–18. For experiments longer than 16 days, we used sharpened tungsten needles to excise optic vesicles to prevent overgrowth and necrosis. Excision was typically carried out from days 10–12. To increase survival and differentiation, 500 nM all-trans retinoic acid (ATRA; #R2625; Sigma-Aldrich, St. Louis, MO, USA) was added to LTR medium from day 20.

## 3. Results

### 3.1. Generation of Inducible High-Fidelity Cas9 PSC Lines

**Figure 1:**
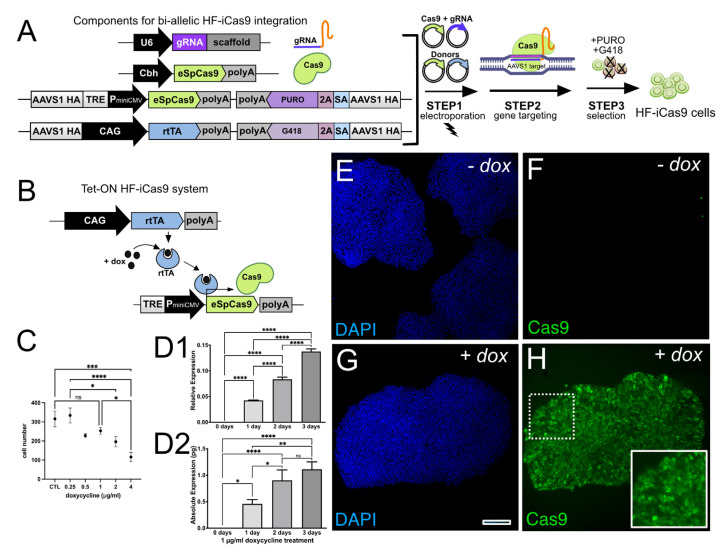
Development of Tet-inducible high-fidelity HF-iCas9 expressing cell lines. A platform for gene editing was devised by generating HF-iCas9 hPSC lines that incorporated a (**A**) plasmid carrying a U6 promoter for transient expression of an AAVS1 guide RNA (gRNA) and eSpCas9, and plasmids for biallelic integration of Tet-inducible eSpCas9 and constitutive reverse tetracycline-controlled trans-activator (rtTA) both at separate alleles at the AAVS1 SHS. A promoter-less splice acceptor (SA) site linked to the p2A sequence allows for the integration of the cassette within the intron of AAVS1 reducing chances of random integration. (**B**) A diagram highlighting the 3G Tet-On system for inducible HF-iCas9 that is activated upon binding of rtTA/dox to a tetracycline response element (TRE). (**C**) Assessment of the influence of dox concentration on cell proliferation. (**D1**,**D2**) Normalized relative (top) and absolute (bottom) expression of Cas9 measured by qRT-PCR after 0 to 3 days of dox (1 μg/mL). At day 0, the expression of Cas9 was undetectable. (**E**–**H**) Immunostaining against Cas9 in PSCs grown in the absence (**E**,**F**) or presence (**G**,**H**) of dox. Nuclei visualized with DAPI alone (**E**,**G**) or together with Cas9 (**F**,**H**). One-way ANOVA was used to test statistical significance and for multiple comparison tests, a Holm-Šídák’s multiple comparison was used with an α cutoff of 0.05. * *p* < 0.05, ** *p* < 0.01, *** *p* < 0.001, **** *p* < 0.0001, ns = not significant. Scale bar (**E**–**H**) = 200 µm. HA = Homology arm.

To produce a set of standardized high-fidelity Tet-On inducible HF-iCas9 expressing cell lines, we modified PSCs from different genetic backgrounds (IMR90.4 and EP1.1 induced pluripotent stem cells (iPSCs) and WA09 embryonic stem cells (ESCs)) by using CRISPR/Cas9 gene editing to insert a third-generation reverse tetracycline trans-activating protein (rtTA) and a Tet response element (TRE) regulated HF-iCas9 both at the AAVS1 (PPRC1) SHS. The TRE was comprised of 7 repeats of a 19-nucleotide tetracycline operator (tetO) sequence and a strong mini-CMV promoter. The sequential use of G418 and puromycin allowed for the efficient ablation of cells that failed to integrate both rtTA and HF-iCas9, respectively (Figure 1A). Bi-allelic targeting resulted in constitutive rtTA expression driven by a CAG promoter and TRE-driven HF-iCas9 expression (Figure 1B). A negative effect on cell growth has been observed with doxycycline [29] and since proliferation impacts homology-directed repair, we wanted to identify a dose where proliferation is not impaired but recombination still occurs. First, we tested doxycycline (dox) up to 2 μg/mL (*n* = 3) and counted the total cell number of cells after 4 days. In a dose-dependent fashion, dox led to decreased cell proliferation (Figure 1C) with 1 μg/mL identified as the lowest dose that did not reduce proliferation; thus, this dose was chosen for subsequent experiments. Next, we evaluated whether the length of dox treatment increased Cas9 mRNA. Using qRT-PCR we observed a time-dependent increase in HF-iCas9 mRNA during the first two days of treatment (Figure 1D). To validate that 1µg/mL of dox treatment produces actual Cas9 protein, we performed immunocytochemistry on clonally selected replica plated iPSC colonies selected for stable integration of Cas9 and rtTA (Figure 1E–H). Replica plating served two functions, first, it validated that Cas9 protein was made, and second, it confirmed whether clones were homogeneous for Cas9 expression. While Cas9 was not detected in untreated samples (Figure 1F), it was highly expressed after dox treatment (Figure 1H) in many, but not all colonies, thus confirming that HF-iCas9 expression is tightly regulated by dox. This approach was taken for the IMR90.4, EP1.1, and WA09 PSC lines. Chromosomal integrity of the unmodified and HF-iCas9 modified cells was analyzed by copy number variation (CNV) analysis, which has a 5-fold greater resolution than standard G-band karyotyping, and no karyotypic changes were observed post-integration of HF-iCas9 in any of the PSC lines (Figure S1).

### 3.2. Efficient Targeting of Transcriptionally Active Endogenous Loci by HDR Using a SOX2-GFP Reporter

**Figure 2:**
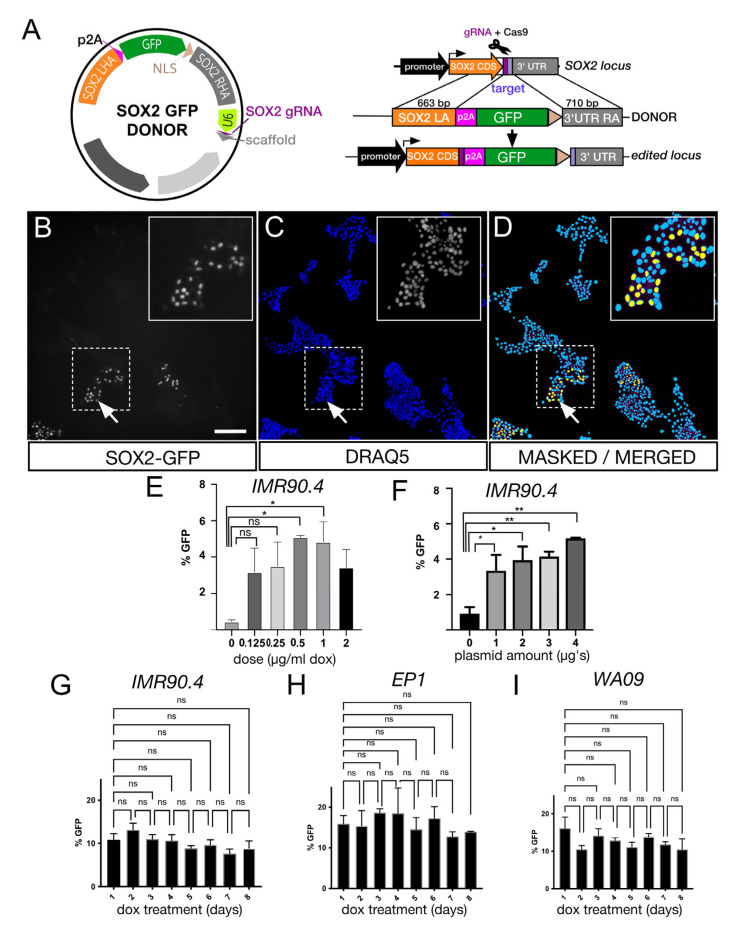
Doxycycline inducible gene targeting at the SOX2 locus. (**A**) A diagram illustrating the design of a hybrid SOX2-GFP donor vector that carries a targeting cassette for the endogenous SOX2 gene and a U6 promoter driving a SOX2 specific guide RNA (left) and an overview of the editing strategy at the SOX2 locus (right). Imaging of SOX2-GFP integrated cells visualized by (**B**) GFP fluorescence and (**C**) DRAQ5 nuclear counterstain fluorescence at 5 days post-transfection. (**D**) GFP+ cells (yellow; arrows) and DRAQ5 labeled nuclei (blue) were identified by automated segmentation and mask generation used for cell counting. (**E**) SOX2-GFP integration efficiency in IMR90.4-HF-iCas9 cells after increasing doses of dox, or (**F**) increasing doses of plasmid for transfection. (**G**) IMR90.4-HF-iCas9, (**H**) EP1.1-HF-iCas9 and (**I**) WA09-HF-iCas9 PSCs following a time course treatment with dox. Each sample was calculated from 25 fields (*n* > 3 biological replicates) of view with error bars represented as standard error of the mean (SEM). One-way ANOVA was used to test statistical significance and for multiple comparison tests, a Dunnett’s multiple comparison procedure was used with an α cutoff of 0.05. * *p* < 0.05, ** *p* < 0.01, ns = not significant. Scale bar (**B**–**D**) = 200 µm.

After establishing that HF-iCas9 was stably integrated and inducible, we next evaluated its effectiveness in mediating HDR at the endogenous SOX2 gene using a SOX2-GFP donor. The single plasmid system developed for this incorporated a U6-driven SOX2 gRNA that targeted the SOX2 translation stop site and a SOX2 DNA donor with flanking left and right homology arms (LHA and RHA) of 633 and 710 base pairs, respectively. A polycistronic translation skipping porcine teschovirus-1 2A (p2A) peptide sequence linked the SOX2 gene in-frame with an enhanced green fluorescent protein (GFP) containing a triple repeated SV40 nuclear localization signal (NLS) (Figure 2A). Linking the GFP with p2A allowed for the separate polycistronic expression of SOX2 and GFP instead of a single fusion protein. Unlike a constitutively expressed fluorescent gene that produces fluorescence soon after transfection, targeting an endogenous gene only leads to fluorescence after in-frame integration with an active gene, in this case, SOX2. Thus, one advantage of targeting SOX2 in PSCs is that since SOX2 is expressed in PSCs, any detection of GFP would indicate gene integration and thereby eliminating the need for labor-intensive genotyping. To optimize gene-editing efficiency, we developed an image-based assay to assess SOX2-GFP integration at 5 days by counting the number of GFP+ and GFP- cells as a percentage of DRAQ5+ cells (Figure 2B–D). After automated image acquisition using an ImageXpress Micro confocal high-content imaging system, images were segmented and analyzed in an unbiased fashion using a custom nuclei counting module in the MetaXpress software package (see Methods). Using this approach, we tested a dox dose–response from 0 to 2 μg/mL to determine the dose necessary to elicit gene editing (Figure 2E). While doses from 0 to 0.25 μg/mL were not significant, doses at 0.5 μg/mL and above were significant (*adj. *p* = 0.0225). Above 1 μg/mL we observed a slight decrease in editing efficiency which we speculate to be a result of the lower cell proliferation that we previously documented. The total amount of plasmid used for transfections was also optimized (Figure 2F), and GFP knock-in was observed from between 1 and 4 μg with a maximal response at 4 μg. Based on these findings, 1 μg/mL dox combined with 4 μg of plasmid DNA was the best suited for gene editing. We next extended this to include different dox treatment lengths which we carried out in 3 different genetic backgrounds to demonstrate that this was not cell line dependent (Figure 2G–I). IMR90.4 (G), EP1.1 (H) and WA09 (I) HF-iCas9 cells were each pre-treated overnight prior to transfection and cells were incubated for an additional 1 to 8 days with dox. All treatments resulted in GFP integration in each genetic background, but there was no statistically significant difference between the number of days of dox treatment and HDR efficiency despite the increase in mRNA we previously observed by qRT-PCR. These results indicate that a short-term dox treatment is sufficient to drive gene-editing and that longer treatments provided no additional benefit.

### 3.3. Optimization of Hybrid Donor Design to Enhance Editing Efficiency

**Figure 3:**
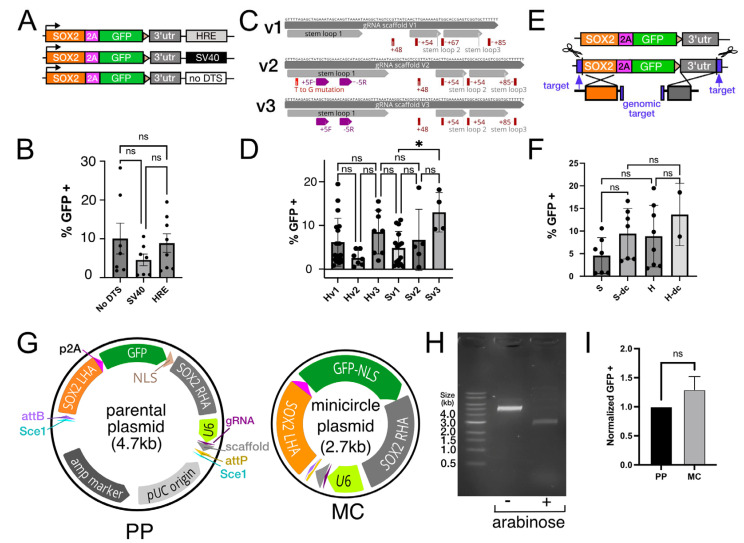
Donor plasmid optimization for gene-editing by homology-directed repair. (**A**) Different DNA tagging sequences (DTS) were inserted into the SOX2-GFP donor plasmids. (**B**) The role of SV40 and HRE DTS sequences on SOX2-GFP integration was assessed by measuring the number of GFP+ cells relative to DAPI+ total cells. (**C**) Three different guide RNA scaffolds based on Ran et al. (2013) [4] (V1), Dang et al. (2015) [33] (V2) and Chen et al. (2013) [34] (V3) illustrate the variation in the first stem-loop structure that interacts with Cas9. (**D**) Quantification of GFP+ cells after editing with SOX2-GFP donor plasmids harboring HRE and SV40 DTSs and the V1-3 scaffolds. (**E**) Illustration of SOX2-GFP donor fragment double cutter (dc) linearization by the introduction of SOX2 targeting sites at the distal ends of the homology arms. (**F**) Quantification of the effect of donor fragment linearization on integration in the presence of SV40 or HRE DTS fragments. (**G**) Illustration of a standard parental plasmid (PP) and a minicircle plasmid (MC) with bacterial sequence elements removed. (**H**) Agarose gel electrophoresis of Kpn1 digested PP and MC plasmids in the absence (−) or presence (+) of arabinose. (**I**) Comparison between PP and MC plasmids for SOX2-GFP integration. One-way ANOVA was used to test statistical significance and for multiple comparison tests, a Dunnett’s multiple comparison was used with an α cutoff of 0.05. * *p* < 0.05, ns = not significant.

Sequence composition has been reported to influence plasmid transfection efficiency and one form of variation includes DNA-targeting sequences (DTS) which are transcription factor binding sequences that are thought to influence plasmid entry into the nucleus by nuclear translocation. These binding sites can augment or inhibit transfection based on the context in which they are used [6,11,30]. To test this we added HRE (hypoxia response element) and SV40 (Figure 3A,B) DTS sequences to the SOX2-GFP donor plasmid and transfected the plasmids into stem cells grown in hypoxia (5%O_2_/10%CO_2_). IMR90.4-HF-iCas9 PSCs maintained in hypoxia were transfected and 5 days later analyzed for GFP fluorescence. Although plasmids with an SV40 DTS yielded fewer GFP+ cells than HRE-containing plasmids the difference was not statistically significant (Figure 3B). Given that plasmids lacking any DTS sequence also resulted in a roughly equivalent number of GFP+ cells relative to HRE plasmids, it does not appear that DTS elements enhanced gene editing under the conditions tested.

After maturation, the native type-II CRISPR gRNA is composed of a 42-nucleotide CRISPR RNA (crRNA) that directs Cas9 to a specific target sequence and an 89-nucleotide tracrRNA that links the cRNA to Cas9. Although the minimal sequence requirement of a single gRNA (sgRNA) is a +48 nucleotide scaffold [3], a longer version of +85 nucleotides is more efficient and more commonly used [8,31,32]. The gRNA scaffold that interacts with wild-type SpCas9 has undergone several iterations to improve specificity and cutting efficiency; however, cutting by different Cas9 variants, particularly newer high-fidelity Cas9 variants, has not been systematically studied. We evaluated SOX2-GFP integration in EP1.1-HF-iCas9 transfected cells using three different gRNA scaffolds herein referred to as V1 [4,26], V2 [33] and V3 [34,35] (Figure 3C,D). In the case of V2 and V3, the first stem-loop structure contains a flip in the fifth nucleotide and an extension to enhance the GAAA loop structure. Overall, no statistically significant difference was noted for the three scaffolds when accompanied by an HRE DTS sequence. We did, however, note a slight increase in SOX2-GFP integration in V3 relative to V1 when accompanied by an SV40 DTS (* adj. *p* = 0.041) (Figure 3D).

Next, we tested whether linearizing a donor plasmid could increase integration. A similar approach has been carried out by introducing gRNA target sites at the distal ends of homology arms within the donor plasmids [36,37]. To verify whether linearizing donor vectors work in HF-iCas9-induced PSCs, we modified the SOX2-GFP donor plasmid to include 23 base pair SOX2 gRNA targets near the distal ends of each homology arm (Figure 3E). Dox-treated EP1.1-HF-iCas9 PSCs were transfected with a standard SOX2-GFP plasmid or a double cutter (dc) HDR donor in the presence of eHRE or SV40 DTS sequences. Despite an elevated average number of GFP + cells in dc samples (Figure 3F), the differences were not significant. While an increase in gene-editing efficiency is possible, the variability between treatment groups and experiments complicated our analysis.

Minicircle technology uses the catalytic activity of øC31-integrase for recombination of attP and attB sites and the I-SceI homing-endonuclease to degrade unwanted bacterial parental plasmid. Specialized minicircle producer *E. coli* have integrated into their genomes an arabinose inducible øC31-integrase, an arabinose transporter LacY A177C gene and the I-SceI homing-endonuclease. Arabinose induction of integrase and endonuclease leads to recombination and a plasmid that is significantly reduced in size. To test whether our hybrid plasmid was minicircle compatible we transformed the SOX2-GFP donor into ZYCY10P3S2T minicircle producer *E. coli* cells in the presence or absence of arabinose. Comparison of Kpn1 digested parental (PP) and minicircle (MC) plasmids revealed 4.7 and 2.7 kb (Figure 3H) size fragments corresponding to the original PP and MC plasmids, respectively (Figure 3G), thereby confirming that our plasmid was minicircle compatible. To confirm that the minicircle can result in gene integration, we transfected it into dox-treated PSCs. After transfection, we saw no significant difference in GFP+ cells between MC and PP plasmid preparations (Figure 3I). Thus, while minicircles have a better safety profile since they are devoid of unwanted bacterial plasmid DNA, their smaller size did not result in higher levels of gene integration into the SOX2 locus.

### 3.4. Gene-Edited SOX2-GFP iPSCs Maintain Their Potential to Differentiate into Neurons

**Figure 4:**
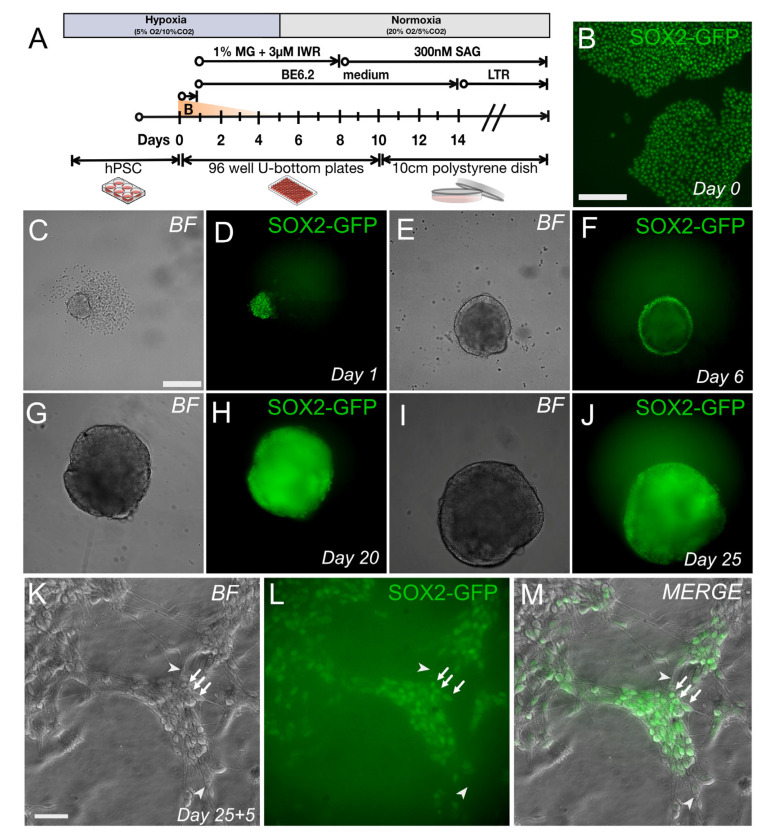
Differentiation potential of gene-edited SOX2-GFP IMR90.4 iPSCs. (**A**) SOX2-GFP integrated organoids were differentiated using a standard retinal organoid differentiation protocol. (**B**) IMR90.4-HF-iCas9 SOX2-GFP PSCs expressing GFP from the endogenous SOX2 gene. (**C**–**J**) Developing organoids imaged in brightfield (BF) (**C**,**E**,**G**,**I**) and SIX6-GFP fluorescence (**D**,**F**,**H**,**J**) monitored from days 1 through day 25. (**K**–**M**) Dissociated day 25 organoids were maintained as a monolayer for 5 days. Arrows indicate SOX2-GFP expression while arrowheads show an absence of fluorescence signal. Scale bars (**B**–**J**) = 200 µm; (**K**–**M**) = 75 µm.

To validate that gene-edited PSCs still retained their ability to form neurons, we carried out 3D organoids differentiation as previously described [22,28] (Figure 4A). IMR90.4 iPSCs with SOX2-GFP expression (Figure 4B) maintained their fluorescence during differentiation up to day 25 (Figure 4C–J). Since it is sometimes difficult to assess fluorescent signals deep within organoids, we dissociated day 25 organoids and cultured them as monolayers on Matrigel-coated TC plates (Figure 4K–M). After 5 more days most neurons had retained their SOX2-GFP expression (arrows), however, some had not (arrowheads). This was expected since SOX2 is known to be expressed in stem cells and neural progenitors, but not in mature cells, other than Müller glia [38]. This data showed that gene-edited HF-iCas9 cells retained their ability to differentiate into early-stage neurons.

### 3.5. Efficient Generation of SIX6 Reporter PSC Lines in Different Genetic Backgrounds

**Figure 5:**
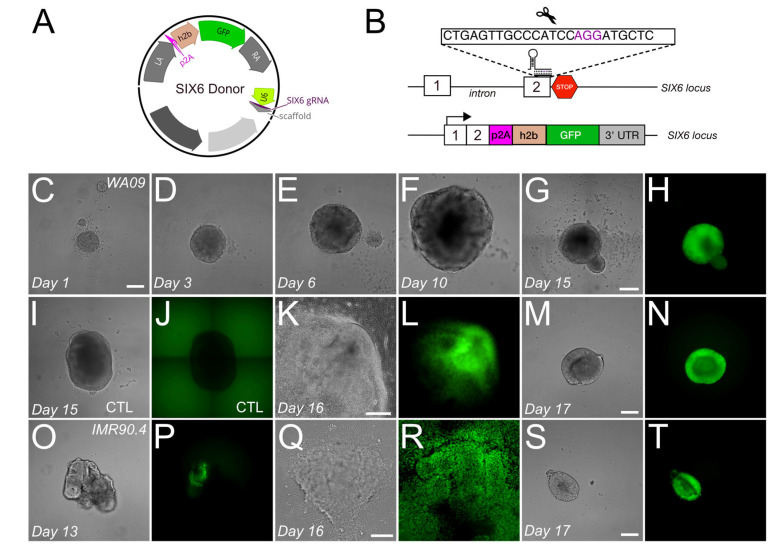
Targeting of the SIX6 gene in multiple genetic backgrounds and differentiation into 3D organoids. (**A**) A SIX6 hybrid donor vector with homology arms flanking the *SIX6* stop codon and a U6 promoter for guide RNA targeting. (**B**) A schematic of SIX6-GFP integration at the *SIX6* stop codon. (**C**–**G**) Brightfield images from days 1–15 organoids in WA09-HF-iCas9 SIX6-GFP ESCs with (**H**) fluorescence detected on day 15. (**I**,**J**) SIX6-GFP negative control organoids from WA09-HF-iCas9 SIX6-GFP ESCs in brightfield and GFP fluorescence. (**K**,**L**) WA09-HF-iCas9 SIX6-GFP organoids derived with an alternative protocol grown as a monolayer and (**M**,**N**) lifted into suspension. (**O**,**P**) IMR90-HF-iCas9 SIX6-GFP organoids under brightfield and fluorescence at day 13 or using an alternative protocol in (**Q**,**R**) adherent culture or (**S**,**T**) lifted into suspension. Scale bars (**C**–**F**,**M**–**P**,**S**–**T**) = 200 µm; (**G**–**J**) = 400 µm; (**K**–**L**,**Q**–**R**) = 150 µm.

While Cas9 can cleave DNA at both methylated and unmethylated sites, HDR is thought to be more sensitive to methylation which might explain the variability observed at gene loci across studies. To determine whether our approach was compatible with HDR at transcriptionally inactive loci we targeted the SIX6 (OPTX2) gene which is silent in stem cells but active during early eye formation. To show that this approach is generalizable across different genetic backgrounds we targeted SIX6 in EP1.1-HF-iCas9, IMR90.4-HF-iCas9 and WA09-HF-iCas9 inducible cells. Like our previous work, we used a p2A-h2b-eGFP (SIX6-GFP) reporter that inserted SIX6-GFP just before the SIX6 stop codon (Figure 5A,B) [28]. After overnight dox, cells were transfected, clonally selected and screened by PCR to obtain clones for each genetic background with mono- or bi-allelic insertion of p2A-h2b-GFP. Amplicons spanning the p2A-GFP inserts were generated from EP1.1-HF-iCas9, IMR90.4-HF-iCas9 and WA09-HF-iCas9 cells and Sanger sequenced. This confirmed the in-frame insertion of SIX6-GFP before the SIX6 stop site (Figure S2A). To rule out the possibility that genetic manipulations adversely affected their differentiation potential, we differentiated PSCs into 3D retinas (Figure 5C–T). Homozygous clones from WA09-HF-iCas9 cells initially formed neural vesicles (Figure 5C–G) followed by expression of SIX6-GFP after 15 days (Figure 5H) that was detected in SIX6-GFP organoids but not in control WA09 cells differentiated without small molecules (IWR and SAG) to initiate retinal induction (Figure 5I,J). These were also differentiated using an alternative retinal organoid differentiation protocol (Figure 5K–N) [39] with similar success. Likewise, we observed SIX6-GFP in differentiating IMR90.4-HF-iCas9 organoids (Figure 5O–T). A major concern when gene-targeting is the potential for off-target mutagenesis which has been reported in vitro and in vivo [40,41]. To rule this out, we Sanger sequenced all clones at the top predicted off-target sequences (Figure S2B,C; Tables S2 and S3). No unwanted mutagenesis at any of the predicted off-target sites was observed. Thus, these experiments demonstrated that the permanent integration of HF-iCas9 into PSCs facilitates HDR at transcriptionally silent loci, occurs with high fidelity, and does not impair their ability to differentiate into optic vesicles.

### 3.6. CLYBL Safe Harbor Targeted Integration of Constitutively Expressed mRuby3 in Differentiated Neurons

**Figure 6:**
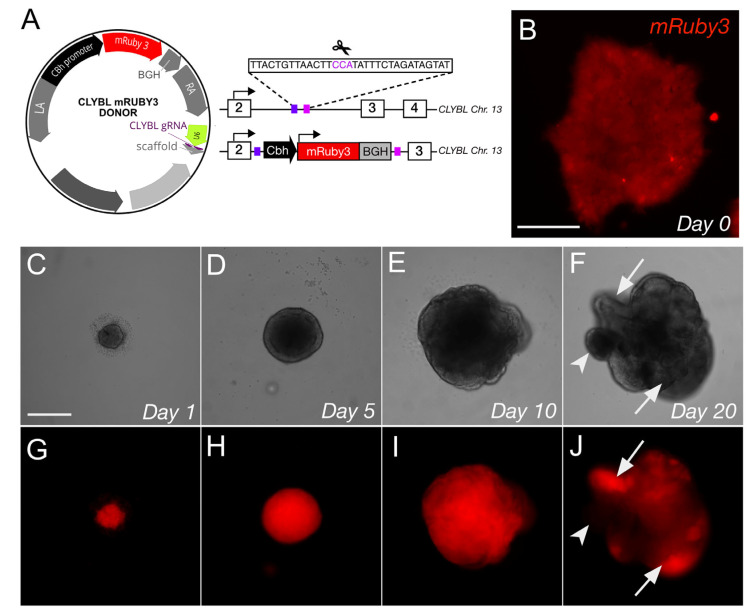
Safe harbor site targeting. (**A**) A donor plasmid (left) for targeting the intronic region (right) of the *CLYBL* SHS contains left and right homology arms (LA, RA), a Cbh promoter, mRuby3 and a BGH polyadenylation sequence. A U6 promoter drives the expression of a *CLYBL*-specific guide RNA for gene targeting. (**B**) Fluorescence of Cbh-mRuby3 integrated at the *CLYBL* SHS in undifferentiated IMR90 iPSCs and imaged by fluorescence microscopy. (**C**–**J**) Organoids derived from IMR90.4-HF-iCas9 CLYBL-mRuby3 PSCs over a 20-day period. (**C**–**F**) Brightfield images were monitored to assess morphology, while (**G**–**J**) constitutively expressed mRuby3 was monitored to assess the preservation of fluorescent signals over time. Arrows = mRuby3 fluorescence, arrowhead = no signal. Scale bars (**B**) = 100 µm, (**C**–**J**) = 200 µm.

Safe harbor sites (SHS) have been utilized for the stable integration of genetic cargo in human cells; however, many studies are limited to the AAVS1 locus. Other sites (e.g., human ROSA locus, H11 and CLYBL) have also been identified but only studied superficially in immortalized cell lines or in PSCs [13,14]. To address this, we used our all-in-one plasmid to test whether the Citrate lyase β-like (CLYBL) site could sustain reporter expression in HF-iCas9 stem cells and differentiating neurons. Cells were dox induced and transfected with a CLYBL-Cbh-mRuby3 (CLYBL-mRuby3) targeting plasmid (Figure 6A) into IMR90.4-HF-iCas9 cells where we observed mRuby3 expression within 24 h. One week later, when transgenes were stably integrated, mRuby3 constitutive expression was evenly distributed in cells that had taken up the transgene. We utilized the presence of mRuby3+ hPSC colonies as a proxy for integration of the transgene cassette instead of Sanger sequencing PCR amplicons. To ensure mRuby3 homogeneity in cells, we clonally passaged PSCs and saw that mRuby3 was qualitatively similar in brightness throughout all clones (Figure 6B). After repeated passage, a loss of fluorescence was occasionally observed in mRuby3+ clones, indicating gene silencing (not shown). To explore whether constitutive mRuby3 PSC reporters maintained expression after differentiation, we differentiated cells into retinal organoids (Figure 6C–J) and observed that CLYBL-mRuby3 PSCs formed stereotypical optic vesicle organoids (Figure 6C–F) that maintained their red fluorescence at early stages (Figure 6G–J, arrows). Not surprisingly, by day 20 some mRuby3 negative pockets had emerged that indicated potential transgene silencing (Figure 6J; arrowhead). Overall, our system works well for CLYBL SHS targeting, however, further work is needed to address potential transgene silencing in different tissue systems.

### 3.7. Mutagenesis by NHEJ

**Figure 7:**
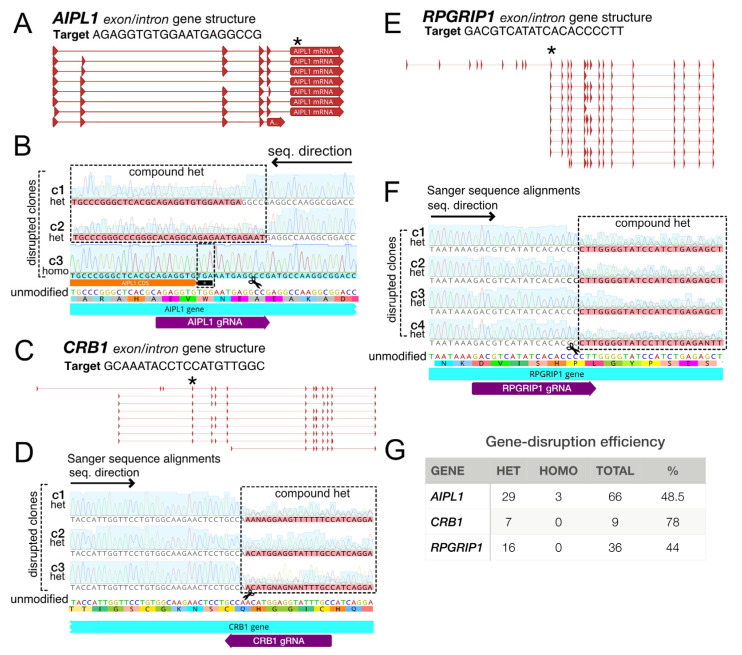
Targeted mutagenesis for generating disease-relevant mutant PSCs. (**A**) Gene organization of the intron/exon structure of the AIPL1 gene and CRISPR targeting location of the final exon near the W278 locus as indicated by an asterisk *. (**B**) Sanger sequence chromatograms (sequenced from right to left) from select clones aligned to unmodified sequences (below) indicate compound heterozygous (c1-2) and homozygous W278X conversion (c3). (**C**) Intron/exon structure of the CRB1 gene and CRISPR targeting at an exon common to 8 out of 9 isoforms. (**D**) Sanger sequence alignments (sequenced from left to right) of the targeted CRB1 locus for several clones (c1-3). (**E**) The RPGRIP1 gene intron/exon structure is displayed with a CRISPR-targeted common exon (*). (**F**) Confirmation of mutagenesis at RPGRIP1 by Sanger sequence alignments (sequenced from left to right) for clones 1–4 (c1-4). (**G**) A table illustrating mutagenesis efficiencies at the AIPL1, CRB1 and RPGRIP1 loci. Efficiency (%) = (heterozygous + homozygous clones/total sequenced clones) * 100. The dotted boxes around alignments highlight gene disruption in various clones while pink shading indicates compound heterozygosity from different reading frames at one or both alleles. Horizontal black arrows indicate the sequencing direction.

To study the effectiveness of gene perturbations in HF-iCas9 stem cells, we targeted several genes linked to Leber congenital amaurosis (LCA), a rare childhood-onset retinal dystrophy caused by mutations in the AIPL1, CRB1, and RPGRIP1 genes, among others. First, we targeted the AIPL1 gene at the final exon near the disease-relevant W278 locus, which is present in the majority of isoforms (Figure 7A). Sanger sequencing confirmed the presence of frame-shift mutations and premature stop codons (Figure 7B). We then targeted the first common exon of CRB1 and RPGRIP1 (Figure 7C–F). Targeting efficiencies ranged markedly, with mutation rates of 48.5%, 78% and 44% for AIPL1, CRB1 and RPGRIP1, respectively (Figure 7G). While AIPL1 targeting resulted in homozygous frame-shift knockout clones, CRB1 and RPGRIP1 only led to heterozygous mutants. With additional colony screening, it is possible that homozygous modifications would also have been obtained at CRB1 and RPGRIP1. Together these results confirmed that in addition to gene knock-in, HF-iCas9 PSCs can be used for efficient loss-of-function by NHEJ.

## 4. Discussion

In the current study, we developed HF-iCas9 integrated cell lines that offer a practical means to generate fluorescent reporter lines and targeted knockouts for disease modeling. HF-iCas9 based on eSpCas9 contains alanine substitutions that weaken interactions between the non-target DNA strand and the HNH/RuvC groove of Cas9, thus preventing strand separation and cutting at off-target sites. The use of HF-iCas9 for gene editing offers the capability to make precision edits with virtually undetectable levels of off-target mutagenesis, thus it is of great interest for cell engineering. One of the bottlenecks for efficient gene editing in PSCs is the generally low levels of HDR which affects reporter generation. Factors contributing to this include inefficient gene delivery, suboptimal gRNA scaffold design, expression levels of Cas9, and synchronization to the cell cycle [42]. In this study, we addressed some of these issues through stable integration of a Tet-inducible HF-iCas9 and developed a single hybrid donor vector that contains a U6-gRNA cassette, an optimized scaffold, a docking site for homology arms, fluorescent gene cargo and is compatible with minicircle technology. While converting plasmids into minicircles did not increase recombination efficiency, the absence of bacterial DNA added a level of safety by reducing the potential for spurious recombination of those elements in PSCs. Eliminating the need for a large Cas9 plasmid and streamlining the donor fragments and U6 cassette into a single hybrid DNA donor also allowed us to add more donor plasmid to transfections which increased gene integration, without excessive cell death.

An enhancer region identified within SV40 DNA, referred to as a ‘DNA nuclear Targeting Sequence’ (DTS), has been shown to improve the transfection efficiency of non-dividing cells [7,30,43,44]. The mechanism is thought to involve the binding of transcription factors (TFs) to DTS elements which interacted with importins to ferry DNA complexes into the nucleus [43]. While reports suggested that SV40 DTSs increased transfection under normoxia, the opposite was true under hypoxia [11]. Conversely, HRE increased transfection efficiency in human 293T cells in vitro and mouse skeletal muscle cells in vivo under hypoxia conditions but has not been evaluated in PSCs. Considering that many labs maintain PSCs under hypoxia, knowledge of the influence of DTS sequences on transfection could be quite helpful. In our hands, HRE and SV40 DTS sequences did not show a statistically significant difference. One possible explanation is that this phenomenon might only work in non-dividing cells or at concentrations within a narrow range above a threshold [45]. In our experiments, we focused on delivering the maximally effective dose so we may have missed this range. It might also be that the hypoxic levels we used (5%O_2_/10%CO_2_) were insufficient for HIF1-α activity. Indeed, the activity of HIF1-α varies greatly at different physiological levels of O_2_, thus future efforts in this area might benefit from a lower range of oxygen levels [13].

Once a gRNA-Cas9 complex forms, a conformational change in Cas9 enables it to bind to double-stranded DNA and cut it. The most common single guide RNA (sgRNA) has a shortened duplex compared with the native bacterial scaffold tracrRNA duplex, which serves as a stop signal for RNA polymerase III. Efforts to modify the sgRNA structure by extending the duplex length and mutating the fourth thymine were reported to dramatically improve knockout efficiency and challenging genome-editing procedures, such as entire gene deletion for loss of function [33]. Contrary to that, HDR at the *SOX2* gene was only marginally improved by altering the guide RNA scaffold. While Dang et al. studied gene knockout, our study focused on HDR, thus we cannot rule out the possibility that the modified stem-loop structure only enhances NHEJ. In addition, their study used a wild-type SpCas9 protein, so it is possible that the modified scaffold does not work as efficiently with our HF-iCas9 as it does with wild-type Cas9. Future work focusing on the differences between HDR and NHEJ in wild-type and high-fidelity Cas9 could shed light on this area. Further, while our system used two separate cassettes and selection markers to generate the Tet-inducible eSpCas9 expressing hPSC lines, a potential direction for simplifying this system would be to incorporate both the rtTA and eSpCas9 into the same plasmid under different promoters to allow for easier screening via a single antibiotic marker. Lastly, it should be noted that other approaches for simplifying gene editing, include the use of Cas9 Ribonucleoprotein (RNP) guide RNA complexes which can be quite efficient. Their main drawback, however, is that their generally higher cost may limit their use in high-throughput applications.

Overall, we developed Tet-inducible high-fidelity HF-iCas9 PSCs with a streamlined gene-editing pipeline involving a simplified hybrid donor that expressed a U6 driven DNA targeting guide RNA (gRNA) and a DNA donor template for targeted gene knock-in or knockout. This platform will accelerate the creation of fluorescent reporters for studies of neural development and mutant cells for disease modeling.

## Data Availability

Plasmids and constructs used in this study are available upon request.

## Supplementary Materials

The following supporting information can be downloaded at: https://www.mdpi.com/article/10.3390/genes13122363/s1, Figure S1: Chromosomal integrity verification of PSCs; Figure S2: Analysis of potential off-target sites for SIX6 targeting and sequence verification; Table S1: Oligonucleotides for vector construction, Table S2: Off-target score analysis; Table S3: Oligos for on-target and off-target genotyping and sequencing; Table S4: Oligos for guide RNA synthesis.
