# Supplementary material for: A Tet-Inducible CRISPR Platform for High-Fidelity Editing of Human Pluripotent Stem Cells
Source: Genes (Basel). 2022 Dec 14;13(12):2363. doi: 10.3390/genes13122363 (PMC9777998; doi:10.3390/genes13122363)
Supplement: Supplementary file 1 [file genes-13-02363-s001.zip › genes-2047481-supplementary.pdf]

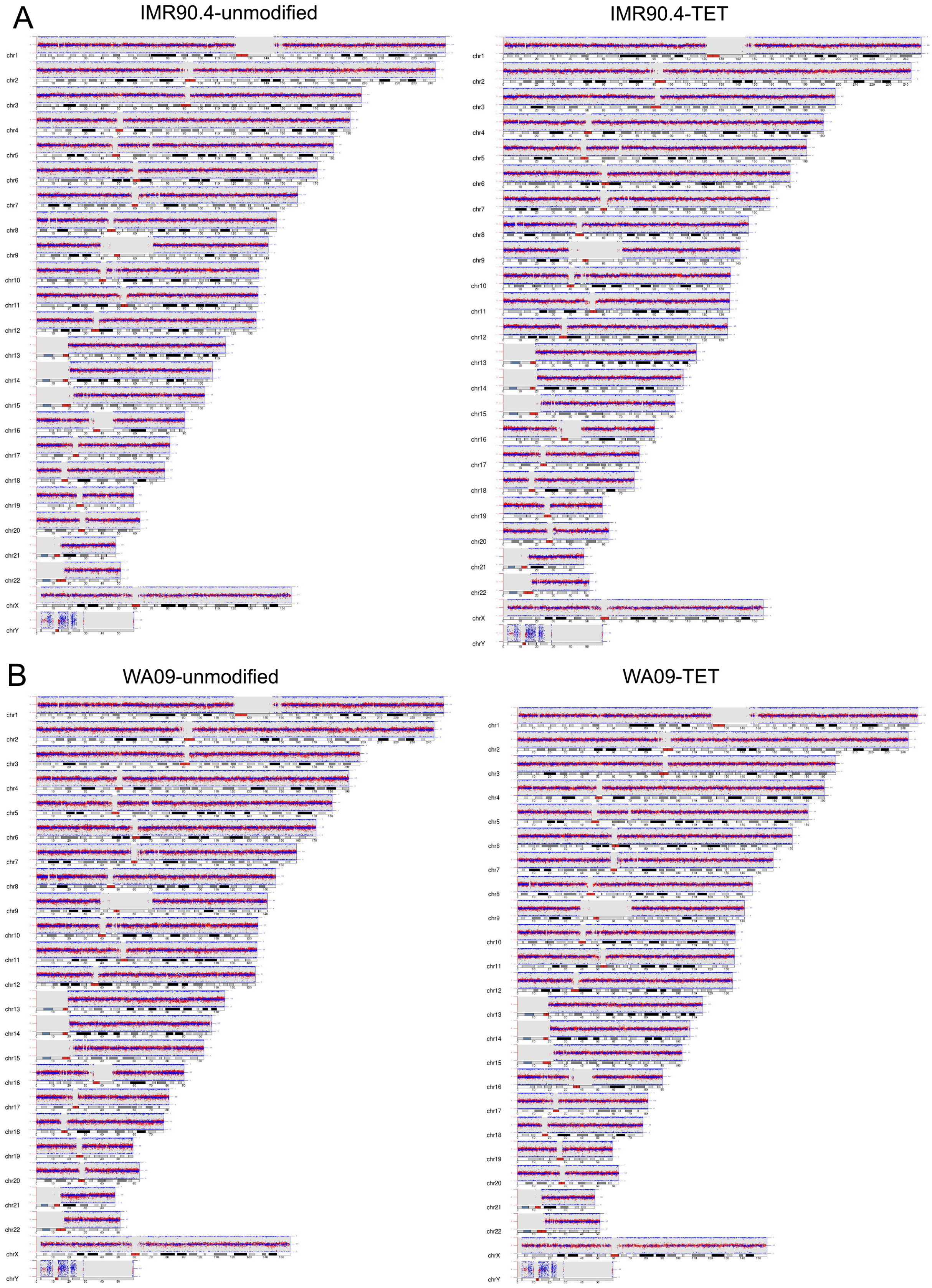
A
IMR90.4-unmodified
IMR90.4-TET
B
WA09-unmodified
WA09-TET
chr1
chr2
chr3
chr4
chr5
chr6
chr7
chr8
chr9
chr10
chr11
chr12
chr13
chr14
chr15
chr16
chr17
chr18
chr19
chr20
chr21
chr22
chrX
chrY

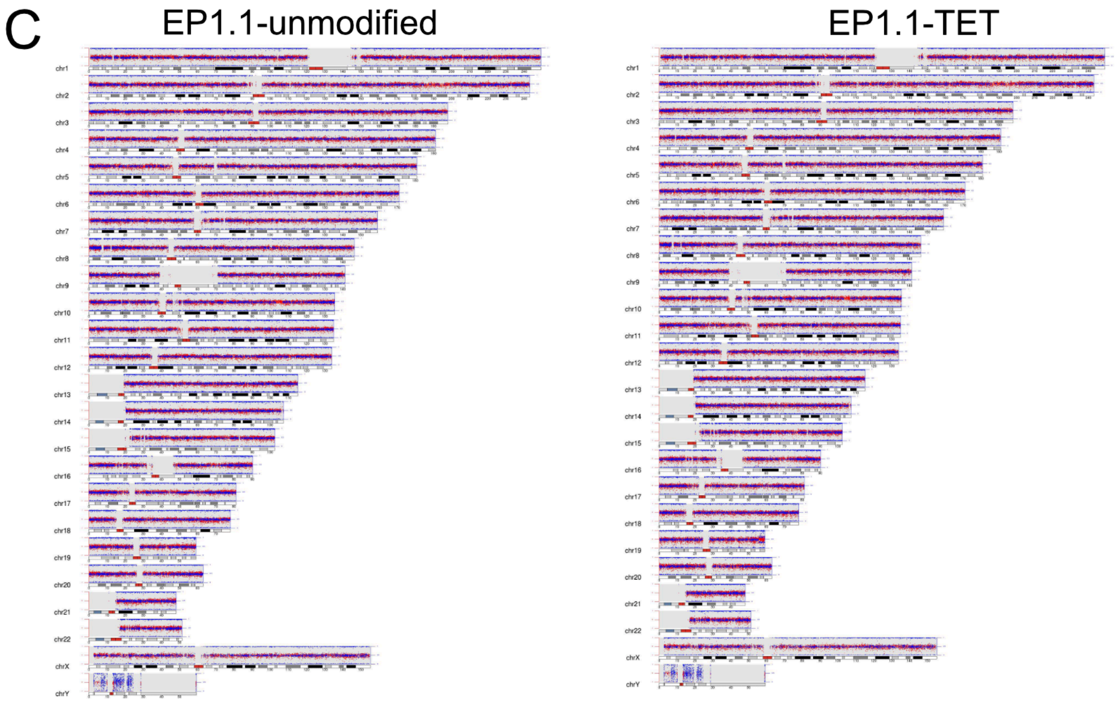

Figure S1. Chromosomal integrity verification of PSCs. Copy number variation (CNV) analyses of unmodified IMR90.4 (A, top left), WA09 (B, center left) and EP1.1 hPSCs (C, bottom left) compared to IMR90.4 (A, top right), WA09 (B, center right) and EP1.1 hPSCs (C, bottom right) following CRISPR-Cas9 gene editing using the TET-inducible platform (right) showcasing no obvious changes post gene editing.

A

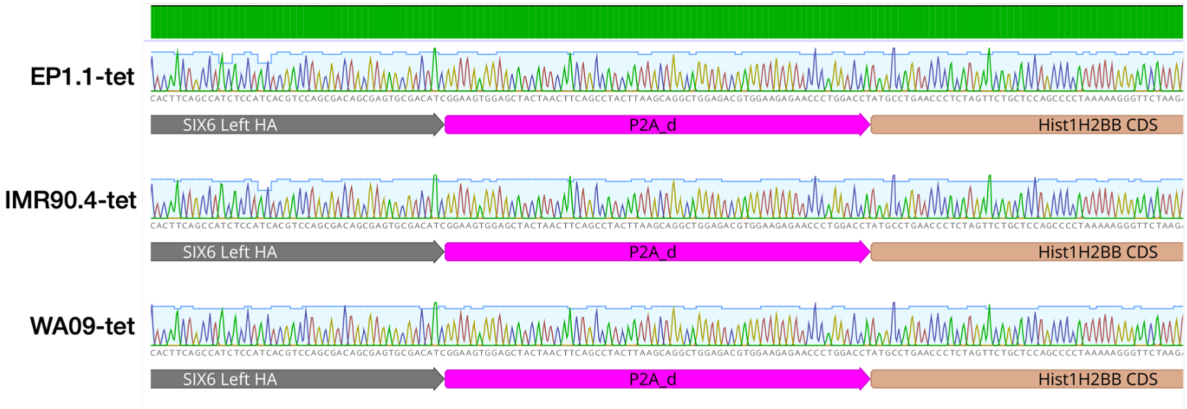

B

Gene	gRNA sequence	off-target score	potential off-target sequence	Chr.	site
**SIX6**	GACATCTGAGTTGCCCATCC	8.69%	AACCTCCGAGTTGCCCATCCTGG	chr1	181,213,353 -> 181,213,375
		1.71%	TACATCTGGGTTGCCAATCCAGG	chrX	10,613,890 <- 10,613,912
		0.58%	GACTTCTGAGTTGGACATCCAGG	chr15	87,452,170 -> 87,452,192

C

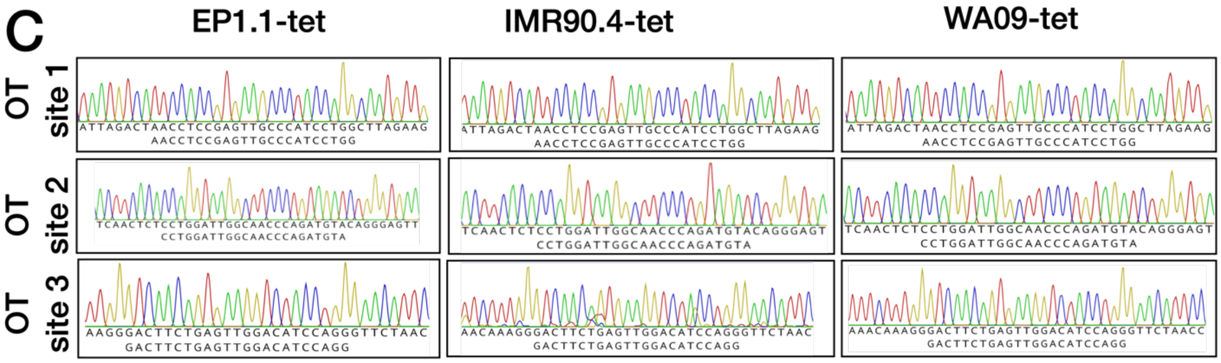

Figure S2. Analysis of potential off-target sites for SIX6 targeting and sequence verification. (A) Sequence verification of in-frame insertion of p2A-H2B-eGFP into the SIX6 locus in HF-iCas9 EP1.1, IMR90.4, and WA09 PSCs. (B) An off-target specificity score predicted using the off-target algorithm from the Zhang Lab at MIT, 2013 shows three potential off-target sites. (C) PCR amplification of the target sites and Sanger sequencing demonstrating a lack of off-target mutagenesis at this site.

Table S1. Oligonucleotides for vector construction.

Name	Sequence
p459_puro_F	TGTCTCAGCTGGGAGGCGACA
p459_puro_R	AGCGCGCAGAGAGGGAGTGG
Cas9backbone_F	GACCTGTCTCAGCTGGGAGGCGACAAAAGGCCGGCGGCCACGAAA
Cas9_R	GGCCGATGCTGTACTTCTTGTCGGCTCTGGACTCCGTGGATACCG
Cas9insert_F	GACAAGAAGTACAGCATCGGCCTGGACATCGGCACCAAC
Cas9insert_R	GTCGCCTCCCAGCTGAGACAGGTCGATCCGTGTCTCGTAC
Cas9insert_R	GTCGCCTCCCAGCTGAGACAGGTCGATCCGTGTCTCGTAC
Cas9insert_F	GACAAGAAGTACAGCATCGGCCTGGACATCGGCACCAAC
MC_2428F	TTTGGTACCAAGCTTTGCGCG
MC_2428R	TATATCTGGCCCGTACATCGAAGCTTTAC
p459_puro_F	TGTCTCAGCTGGGAGGCGACA
p459_puro_R	AGCGCGCAGAGAGGGAGTGG
hU6prom_F	AGGCGCCTAGGGAATTCGCGGCCGCCTTTTGCTCACATGTGAGGGC
hU6prom_rev	TTACCCCAGTTGGGGTCGCGGCCGCCAAAAAAGCACCGACTCGGT
Cas9backbone_F	GACCTGTCTCAGCTGGGAGGCGACAAAAGGCCGGCGGCCACGAAA
Cas9backbone_R	GGCCGATGCTGTACTTCTTGTCGGCTGCTGGGACTCCGTGGATACCG
eHRE_F	CGTGTACGTGTGTATGTGAAGATACCGCCTAGGCGCCTAGGGAATTCGCG
eHRE_R	TTCACATACACACGTACACGCACACACATACATCCGCGCAAAGCTTGGTACCAAA
Cbh_Mlu1_H11oh_F	GGAAAAAGGCCATAGTTGATACGCGTGCTCTAGAGGTACC
Cbh_kozak_Ruby3ohR	CCCTTAGACACCATGGTGGCCCAACCTGAAAAAAAGTGAT
Ruby3_kozak_F	GCCACCATGGTGTCTAAGGGC
H11_LA_R	ATCAACTATGGCCTTTTTCCTTGAGCTTTA
mcSOX2_F	CTGCGAGCGCTGCACATGAA
mcSOX2_R	AGACCACAGAGATGGTTCGCCAGT

Table S2. Off-target score analysis.

Gene Symbol ID	OT score	#OT sites	Target Sequence	Off-target Sequence	position
**AAVS1 PPP1R12C ID: 54776**	97.85%	3 (0 in CDS)	GTCACCAATCCTGTCCCTAG *TGG*		Chr19
	5.45%			G**C**CACCA**C**TCCTGTCCCT**G**GTGG	chr15 NC_000015) 71,357,378 -> 71,357,400
	3.30%			G**C**CAC**A**AATCCTGTCCCT**G**G**A**GG	chr16NC_000016) 55,430,764 -> 55,430,786
	2.22%			G**C**CACCA**C**TCCTG**G**CCCTAGTGG	chr15 NC_000015) 50,552,119 -> 50,552,14
**CLYBL ID:171425**	89.11%	17 (0 CDS)	GACCATACTATCTAGAAATA *TGG*		
	7.34%			G**G**C**A**A**A**ACTATCTAGAAATATGG	chr2 NC_000002 235,341,428 <- 235,341,450
	7.01%			**A**A**A**CATACTAT**A**TAGAAATATGG	chr17 NC_000017 40,233,455 <- 40,233,477
	5.89%			GAC**TT**TACTATCTAGA**G**ATA**G**GG	chrX NC_000023) 96,166,767 <- 96,166,789
	5.75%			**A**ACCATA**G**TATC**C**AGAAATA**G**GG	chr4 - NC_000004) 55,964,545 -> 55,964,567
	5.20%			GA**G**C**T**TACTATCTAGAAA**G**A**G**GG	chr2 - NC_000002) 21,428,393 <- 21,428,415
**SIX6 ID: 4990**	97.85	3 (0 in CDS)	GACATCTGAGTTGCCCATCC *AGG*		Chr14
	8.69%			**A**AC**C**TC**C**GAGTTGCCCATCC***T****GG*	chr1 NC_000001) 181,213,353 -> 181,213,375
	1.71%			***T****ACATCTG****G****GTTGCC****A****ATCCAGG*	chrX NC_000023) 10,613,890 <- 10,613,912
	0.58%			**G**ACTTCTGAGTTG**GA**CATCC*AGG*	chr15NC_000015)

					87,452,170 -> 87,452,192
**SOX2 ID: 6657**	86.26%	5 (0 in CDS)	GGCCCTCACATGTGTGAGAG *<u>GGG</u>*		Chr3
	26.8			GGCCCTCACATGTG**C**GAGAGGGG	chr8 NC_000008) 124,301,810 -> 124,301,832
	21.25%			G**A**CCCTCACAT**A**TGTGAGAG**A**GG	chr12 106,588,407 -> 106,588,429
	12.13%			**TA**CC**T**TCACATGTGTGAGAG**T**GG	chr15 92,650,877 <- 92,650,899
	7.01%			**A**G**T**CCTCACAT**A**TGTGAGAG**T**GG	chr14 27,884,782 <- 27,884,804

OT score – off target specificity score
Underlined Italics = PAM sequence
grey background = target sequence different than query

Table S3. Oligonucleotides for on-target and off-target genotyping and sequencing. Genotyping primer pairs end with 'F' and 'R' whereas sequencing oligonucleotides end with 'Seq' or 'Seq_F' or 'Seq_R'.

Gene	Oligo name	Sequence	Amplicon Size
**AAVS1**	AAVS1_893_OT1_F	ACTTTGGGAGGCTGAGGCAAGAAGATTG	893
	AAVS1_893_OT1_R	TCTTCCTTCTGAACTCTTCCCACAGGGATT	-
	AAVS1_Seq_OT1	TTCCCACTTCCTCATTTCTACTTC	N/A
	AAVS1_904_OT2_F	ACCCTCACCAGCACTGGAAATTGAAAA	904
	AAVS1_904_OT2_R	TTGGGTTTCTCTCCTCTAGCATGGATCTC	-
	AAVS1_Seq_OT2	CCCATACTTTCTTGGGTAATGTTC	N/A
	AAVS1_861_OT3_F	CCAAGGGCTCTTCAGTCAGGCTCTTACA	861
	AAVS1_861_OT3_R	ATGGAGGGAGCATTTGGACCAGACCTA	-
	AAVS1_Seq_OT3	AATGCTGTTCAAGAGCTAAGTCCT	N/A
	AAVS1_890_OT4_F	TGTCCTGAGTTACAAGGGTGGACAGCAC	890
	AAVS1_890_OT4_R	TTCTTTGTGCCAAGCCCCATGTTAGGTA	-
	AAVS1_Seq_OT4	TGGAGAAGTCTAGTGCTCACATTC	N/A
	AAVS1_876_OT5_F	TGGTGCAATCATGGCTCACTACAGCTTC	876
	AAVS1_876_OT5_R	TCCCTTCCCCCAGGTTAGATAATGTTTTGG	-
	AAVS1_Seq_OT5	AGTAGAGGCGAGGTCTCACTATGT	N/A
**SIX6**	hSIX6GenoT_FlankF	GCAACCGGACTGACCCCTAC	1,831
	hSIX6GenoT_int_R	AGCTAGTGTACTTGGTAACTGCCTTAGTGC	-
	hSIX6_GenoT_2763_F	GCAACCGGACTGACCCCTAC	2,763/3936 *
	hSIX6_GenoT_2763_R	TCGAGGCCATTACCAGGACA	-
	G1_SIX6T1_ot1GT_713F	GCAGTGGGCAAACTGCCTCCA	713
	G2_SIX6T1_ot1GT_713R	GCTCCCTCCTGCTCTTCCTCACC	-
	G3_SIX6T1_ot2GT_803F	GAGACTCCAGTGCAGCCACATGGT	803
	G4_SIX6T1_ot2GT_803R	TGGCAGTGTGGTTCGGGGAGT	-
	G5_SIX6T1_ot3GT_812F	TGTCTTTTGGCTTTCAGGCATGTGG	812
	G6_SIX6T1_ot3GT_812R	AGGGCAGCTTCCAACAGAGAGCAAG	-
	S216_S6_ot1_Seq3_F	AGGGCAGGGAATAAGCAAAT	N/A

S218_S6_ot2_2Seq_F	GCTGAATTGGTCACAGCTCA	N/A
S220_S6_ot3_Seq3_F	CATAAGCCAAAAGGCTGCTT	N/A

*Indicates sizes for no insertion/insertion, respectively.

Table S4. Oligonucleotides for guideRNA synthesis.

Gene	Oligo name	Sequence
**AAVS1**	AAVS1_esp_T1F	CACCGTCACCAATCCTGTCCCTAG
	AAVS1_esp_T1R	AAACCTAGGGACAGGATTGGTGAC
	AAVS1_esp_T2F	CACCGTGGCCCCACTGTGGGGTGG
	AAVS1_esp_T1R	AAACCCACCCCACAGTGGGGCCAC
**CLYBL**	CLYBL gRNA T1 F	GACCATACTATCTAGAAATAGTTTTAGAGCTAGAAATAGCAAGTT
	CLYBL gRNA T1 R	TATTTCTAGATAGTATGGTCGGTGTTTCGTCCTTTCCACA
**SIX6**	SIX6guide_F	TTTCTTGGCTTTATATATCTTGTGGAAAGGACGAAACACC**G**ACATCTGAGTTGCCCATCC
	SIX6guide_rev	GACTAGCCTTATTTTAACTTGCTATTTCTAGCTCTAAAAC GGATGGGCAACTCAGATGT C
**SOX2**	SOX2gRNAswapT2R	CCTCTCACACATGTGAGGGCCGGTGTTTCGTCCTTTCCAC
	SOX2gRNAswapT2F	GGCCCTCACATGTGTGAGAGGTTTTAGAGCTAGAAATAGCAA
